# STAT3 and STAT6 activation in lung tissues correlates with inflammatory and clinical traits in COPD

**DOI:** 10.1183/23120541.01310-2025

**Published:** 2026-03-16

**Authors:** Marco Contoli, Daniela Miglietta, Paolo Casolari, Elisa Schiavi, Debora Fragni, Umberto Semenzato, Federico Baraldi, Simonetta Baraldo, Alberto Papi

**Affiliations:** 1 Section of Respiratory Diseases, Department of Translational Medicine, University of Ferrara, Ferrara, Italy; 2 Azienda Ospedaliera Universitaria Ferrara and AUSL, Ferrara, Italy; 3 Department of Experimental Pharmacology and Translational Science, Chiesi Farmaceutici SpA, Parma, Italy; 4 Department of Cardiac, Thoracic, Vascular Sciences and Public Health, University of Padova and Padova City Hospital, Padua, Italy

## Abstract

COPD is a heterogeneous respiratory disease caused mainly by cigarette smoking in Western countries. COPD is usually progressive and significantly impacts patients’ quality of life. Breathlessness, cough and sputum production are the most common symptoms, and can acutely exacerbate into potentially fatal events. A chronic inflammatory process underlies the development and progression of the disease [1].

## Shareable abstract

**The study highlights the role of JAK/STAT signalling in COPD, with STAT3 linked to neutrophilic inflammation and STAT6 to eosinophilic inflammation and chronic bronchitis. Findings support targeted therapies to address specific clinical traits in COPD.** https://bit.ly/4qoEs0h

*To the Editor:*

COPD is a heterogeneous respiratory disease caused mainly by cigarette smoking in Western countries. COPD is usually progressive and significantly impacts patients’ quality of life. Breathlessness, cough and sputum production are the most common symptoms, and can acutely exacerbate into potentially fatal events. A chronic inflammatory process underlies the development and progression of the disease [1].

Several inflammatory pathways in COPD involve cytokines that signal *via* receptors coupled to the Janus kinase (JAK)/signal transducer and activator of transcription (STAT) pathway [2]. Activation of JAK/STAT pathways has been documented in COPD but the data are limited and their role has not been fully elucidated [3, 4]. In particular, it has not yet been fully investigated whether JAK/STAT activation correlates with specific clinical and/or inflammatory traits.

In mammals, seven STAT isoforms have been identified (STAT1, STAT2, STAT3, STAT4, STAT5A, STAT5B and STAT6), each with distinct roles in cellular responses to various signalling. Here, we focused on STAT pathways in relation to the pathological/clinical features of COPD, including inflammatory profile (type 2 (T2) *versus* T1) and exacerbation risk. This study examined STAT1 (a major mediator of the cellular response to interferons and a key component of the immune response against viruses [5]), STAT3 (mainly activated by interleukin (IL)-6, and essential for regulating inflammation and protease activation [6]), and STAT6 (primarily activated by IL-4 and IL-13, mediating the T2 immune response, airway eosinophilia, epithelial mucus production and smooth muscle changes [7]).

We studied: 1) the activation of the JAK/STAT pathway by measuring the expression of phosphorylated STAT1, STAT3 and STAT6 (pSTATs) in lung tissue from COPD patients compared to controls; and 2) the relationship between the expression of pSTAT family members in lung tissue, and systemic inflammatory markers and clinical traits of COPD, including lung function and chronic bronchitis. Lung tissues were collected from subjects undergoing lung resection for a solitary peripheral nodule, as previously described [8]. Ethics approval was obtained for lung tissue collection and inflammatory profile analysis (Comitato Etico Azienda Ospedaliero Universitaria Ferrara, Ferrara, Italy; ref. number 080399). pSTAT expression was evaluated in the nuclei of both epithelial cells of peripheral airways and alveolar macrophages in the surrounding lung parenchyma by immunohistochemistry (IHC) using commercially available antibodies (anti-STAT1 when phosphorylated at tyrosine 701, Invitrogen catalogue number MA5-15071; anti-STAT3 when phosphorylated at tyrosine 705, Cell Signaling Technology catalogue number BK4113S; anti-STAT6 when phosphorylated at tyrosine 641, Invitrogen catalogue number 700247). EG2 staining (Diagnostic Developments, Uppsala, Sweden) was used to evaluate activated, eosinophil cationic protein-secreting eosinophils [9]. COPD patients were grouped as eosinophil high *versus* eosinophil low or neutrophil high *versus* neutrophil low, based on the median value of blood eosinophil or neutrophil count, respectively. Comparisons among groups were evaluated with either Mann–Whitney test or the Kruskal–Wallis test followed, when results were significant, by Dunn's multiple comparisons test, as appropriate. Correlation coefficients were calculated using Spearman's rank method. A p-value of ≤0.05 was considered to indicate statistical significance.

Based on prior studies [4, 10], 17 COPD patients, 17 smokers with normal lung function (S), and 12 nonsmoking subjects with normal lung function (NS) were included in the analyses. The three groups were comparable in age (mean±sem 71±1.9 *versus* 70±1.1 *versus* 70±1.7 years, respectively) and gender (male: 82% *versus* 82% *versus* 67%). As expected, post-bronchodilator forced expiratory volume in 1 s was lower in COPD (68±2% predicted; p<0.01) compared to S and NS (100±4 *versus* 101±5% predicted). 35% of COPD patients and 29% of S had clinician-confirmed chronic bronchitis (*i.e.* mucus-producing cough occurring on most days for ≥3 months of the year for two consecutive years, with other causes for the cough ruled out [1]). Only one patient in the NS group had chronic bronchitis symptoms. 14 out of the 17 COPD patients were treated with bronchodilators and three were on an inhaled corticosteroid/long-acting β_2_-agonist inhaled regimen.

IHC analysis of pSTAT expression in lung samples revealed significantly higher pSTAT3 expression in both epithelial cells (p<0.01) and alveolar macrophages (p<0.001) of COPD patients compared to NS (figure 1a). A significant increase in pSTAT3 expression was also observed in the epithelial cells (p<0.05) of S compared to NS (figure 1a). Conversely, pSTAT6 was activated in COPD patients compared to the other two groups, with no differences between NS and S (figure 1b). In particular, in COPD samples, pSTAT6 was significantly increased in both epithelial cells (p<0.001) and alveolar macrophages (p<0.05) compared to NS and S (both p<0.01), indicating that pSTAT6 activation occurs in the context of COPD and not merely from tobacco smoke exposure (figure 1b). No difference was found in pSTAT1 expression among the three groups (data not shown). Confirmatory analysis by western blotting was not performed because only paraffin-embedded samples were available.

**FIGURE 1:**
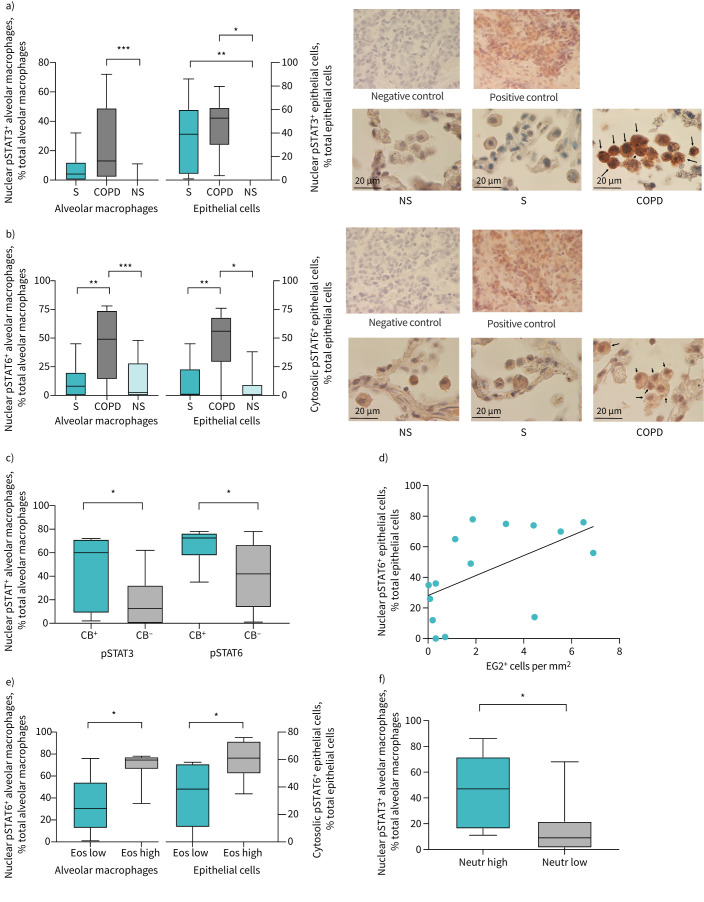
a) Immunohistochemistry (IHC) quantification of phosphorylated signal transducer and activator of transcription (pSTAT)3 in alveolar macrophages and epithelial cells of peripheral airways of COPD patients, smokers with normal lung function (S) and nonsmoker subjects with normal lung function (NS); and representative IHC staining of pSTAT3 in alveolar macrophages of COPD patients, S, NS, and positive and negative controls (breast cancer). b) IHC quantification of pSTAT6 in alveolar macrophages and epithelial cells of peripheral airways of COPD patients, S and NS; and representative IHC staining of pSTAT6 in alveolar macrophages of COPD patients, S, NS, and positive and negative controls (breast cancer). c) IHC quantification of pSTAT3 and in alveolar macrophages of COPD patients with chronic bronchitis (CB) compared to COPD patients without. d) Correlation between IHC quantification of pSTAT6 in alveolar macrophages and the number of EG2^+^ eosinophils in the peripheral lung. e) IHC quantification of pSTAT6 in alveolar macrophages and epithelial cells of peripheral airways of COPD patients with blood eosinophils counts above (Eos high) or below (Eos low) the median value of the study population. f) Expression by immunohistochemistry of pSTAT3 in alveolar macrophages of COPD patients with blood neutrophil counts above (Neutr high) or below (Neutr low) the median value of the study population. *: p<0.05; **: p<0.01; ***: p<0.001.

We found no correlations between pSTAT expression and lung function (data not shown).

Higher pSTAT3 and pSTAT6 expression levels were found in the alveolar macrophages of COPD patients with chronic bronchitis compared to COPD patients without chronic bronchitis (p<0.05) (figure 1c). Interestingly, a correlation was found between pSTAT6 expression (but not pSTAT3) in alveolar macrophages and EG2^+^ cells in peripheral lung of COPD patients (p<0.05, r=0.56) (figure 1d). Consistently, COPD patients with higher blood eosinophil count (median value of the COPD study population 156 cells per µL) had significantly higher pSTAT6 expression (but not pSTAT3) in both epithelial cells and alveolar macrophages (p<0.05) (figure 1e),

Conversely, COPD patients with higher blood neutrophil counts (median value of the COPD study population 7360 cells per µL) had higher pSTAT3 expression in alveolar macrophages compared to those with neutrophil counts below the median (figure 1f). Blood eosinophil and neutrophil counts were weakly but statistically inversely related in COPD patients (p=0.05, r= −0.39; data not shown).

Overall, we confirmed the activation of STAT3 in the lungs of COPD patients, as previously observed by other groups [3–4, 10], and found that pSTAT3 activation is particularly prominent in patients with high blood neutrophils, in line with prior observations of an increased JAK/STAT3 signature in COPD patients with high blood neutrophil counts [10, 11]. Interestingly, pSTAT3 was also increased, though not significantly, in the epithelial cells of S, which is consistent with STAT3 activation observed in animal models [12]. At variance with Yew-Booth *et al*. [4], we did not find differences in pSTAT1 expression in the airways of COPD patients compared to S and NS. Possible explanations for this discrepancy include: 1) different tools of analysis (IHC *versus* western blot) and 2) differences in patient severity. Indeed, in their study, Yew-Booth *et al*. [4] investigated samples from lung transplant patients, while our samples were from milder patients undergoing resection. We report STAT6 activation in COPD lung tissues, particularly in patients with higher blood eosinophil counts and activation of eosinophilic inflammation in peripheral lung, showing an association of pSTAT6 with T2 inflammation in COPD. This finding is in line with recent evidence of the efficacy of targeting the IL-4/T2 pathway in a subset of COPD patients with T2 inflammation [13]. Additionally, higher levels of both pSTAT3 and pSTAT6 were found in COPD patients with concomitant chronic bronchitis, consistent with evidence that: 1) chronic bronchitis is associated with increased airway inflammatory mediators, including eosinophils [14]; and that 2) IL-13/STAT6 pathways are involved in mucus production mechanisms [15].

Our data indicate the involvement of the JAK/STAT pathway in COPD and suggest the potential for novel, tailored pharmacological approaches.
